# Cognitive Assessment in Glaucoma Care: A Narrative Review of Clinical Rationale, Screening Tools, and Implementation Challenges

**DOI:** 10.7759/cureus.108458

**Published:** 2026-05-07

**Authors:** Yuto Yoshida, Masaki Tanito

**Affiliations:** 1 Department of Ophthalmology, Shimane University Faculty of Medicine, Izumo, JPN

**Keywords:** alzheimer’s dementia, dementia care, glaucoma practice, glaucoma therapy, japanese geriatrics

## Abstract

In recent years, increasing attention has been directed toward the association between glaucoma and cognitive impairment, with a growing number of reports highlighting their interrelationship. With the progression of population aging, the coexistence of these two conditions is expected to rise further. Cognitive impairment may influence glaucoma management in multiple ways, including difficulties in performing subjective examinations such as visual field testing, reduced adherence to topical medications, and an elevated risk of postoperative complications. Consequently, integrating cognitive assessment into glaucoma care may be particularly relevant in selected patient populations, such as older adults or those with suspected adherence or reliability issues.

This narrative review aims to summarize the current evidence on the relationship between glaucoma and cognitive impairment and to provide an overview of commonly used cognitive screening tools. In addition, we discuss the clinical implications of cognitive dysfunction in glaucoma management and highlight emerging digital approaches for cognitive assessment. Given that much of the available evidence is observational and that glaucoma-specific validation of cognitive screening strategies remains limited, this review provides insights into optimizing glaucoma care in an aging society and outlines directions for future research.

## Introduction and background

Glaucoma is one of the leading causes of blindness worldwide [1,2]. In recent years, increasing attention has been directed toward the association between glaucoma and dementia, including cognitive impairment. The relationship between glaucoma and dementia is considered to involve complex interactions among multiple factors [1-3].

Glaucoma and cognitive impairment share common risk factors and biological features, including aging, oxidative stress, amyloid-β accumulation, and neuroinflammation [4]. Beyond these shared mechanisms, glaucoma itself may be associated with cognitive decline through both direct and indirect pathways, such as optic nerve degeneration leading to brain atrophy, as well as functional visual loss resulting in depression and social isolation, although these mechanisms remain hypothetical. Recent epidemiological studies have reported that glaucoma is associated with an increased risk of dementia, including Alzheimer’s disease and vascular dementia [5,6]. Conversely, cognitive impairment may hinder glaucoma management by reducing treatment adherence and the reliability of ophthalmic testing [7-10], thereby potentially accelerating disease progression.

Despite these interrelationships, cognitive function is rarely evaluated in routine glaucoma care. Cognitive status can substantially influence clinical decision-making, from the interpretation of perimetric results to ensuring adherence to topical therapy and postoperative care. However, a comprehensive synthesis of current knowledge is lacking. Therefore, this narrative review aims to summarize the current evidence on the relationship between glaucoma and cognitive impairment, with a focus on the clinical impact and assessment strategies. In addition, we provide an overview of conventional and emerging cognitive screening tools, including digital application-based approaches, and discuss their potential role in optimizing glaucoma management in an aging society.

## Review

Methods

This narrative review was conducted to summarize the current evidence on the association between glaucoma and cognitive impairment and its clinical implications. A literature search was performed using PubMed to identify articles published up to April 10, 2026. The search strategy included combinations of the following keywords: “glaucoma,” “cognitive impairment,” “dementia,” and “Alzheimer’s disease.” We primarily included observational studies, systematic reviews, and meta-analyses that examined the relationship between glaucoma and cognitive impairment (Table 1). Additional relevant articles were identified through manual screening of reference lists. Given the narrative nature of this review, study selection was not conducted according to a predefined protocol or PRISMA guidelines. Therefore, the findings should be interpreted in light of potential selection bias and heterogeneity among the included studies.

**Table 1:** Summary of key studies examining the relationship between glaucoma and cognitive impairment AD: Alzheimer’s disease; MCI: mild cognitive impairment; POAG: primary open-angle glaucoma; PACG: primary angle-closure glaucoma; EXG: exfoliation glaucoma; Mini-Cog: Mini-Cognitive Assessment Instrument; MMSE: Mini-Mental State Examination; RNFL: retinal nerve fiber layer; OAG: open-angle glaucoma; Apo: apolipoprotein; TTR: transthyretin; OR: odds ratio; RR: relative risk; CI: confidence interval

Study	Design	Sample size	Glaucoma type	Cognitive factor	Main findings
Wang et al. (2024) [5]	Systematic review & meta-analysis	27 studies; 9,061,675 individuals	Mixed glaucoma	Dementia (all-cause, AD, vascular), MCI	Glaucoma was associated with an increased risk of all-cause dementia (odds ratio (OR) 1.21, 95% CI 1.13-1.29), AD (OR 1.19, 95% CI 1.10-1.29), vascular dementia (OR 1.25, 95% CI 1.09-1.44), and MCI (OR 1.36, 95% CI 1.14-1.61)
Huh et al. (2023) [6]	Meta-analysis	18 studies; 4,975,325 individuals	POAG, PACG	Dementia (all-cause, AD)	POAG was associated with a 29% increased risk of AD (relative risk (RR) 1.29, 95% CI 1.16-1.44)
Tanito et al. (2023) [8]	Cross-sectional study	60 patients	Mixed glaucoma	Cognitive function (Mini-Cog)	Lower cognitive function was significantly associated with eye drop instillation failure (OR 2.7 per score decrease, p = 0.025)
Ichitani et al. (2023) [10]	Cross-sectional study	746 patients	Mixed glaucoma	Cognitive function (Mini-Cog)	Cognitive impairment was significantly associated with higher false negative (p = 0.0034) and false positive rates (p = 0.0051)
Yoshikawa et al. (2021) [11]	Cross-sectional study	172 patients	Mixed glaucoma	Cognitive function (MMSE)	Severe glaucoma was associated with increased odds of cognitive impairment (OR 2.62, 95% CI 1.006-6.84; p = 0.049), and RNFL thinning was also associated with higher risk (OR 1.42 per 10-μm decrease, 95% CI 1.05-1.93; p = 0.025)
Inoue et al. (2013) [12]	Cross-sectional study	90 patients	POAG, EXG	AD-related biomarkers (apolipoprotein (Apo) AI, ApoE, transthyretin (TTR))	OAG eyes showed significantly higher levels of multiple Alzheimer’s disease-related biomarkers

Epidemiology of glaucoma and cognitive impairment

The number of studies focusing on the association between glaucoma and cognitive impairment has increased in recent years. Patients with glaucoma may be at increased risk of cognitive impairment. A previous meta-analysis demonstrated a significant association between glaucoma and cognitive impairment, including all-cause dementia (odds ratio (OR) 1.21, 95% confidence interval (CI) 1.13-1.29), Alzheimer’s disease (OR 1.19, 95% CI 1.10-1.29), vascular dementia (OR 1.25, 95% CI 1.09-1.44), and mild cognitive impairment (MCI) (OR 1.36, 95% CI 1.14-1.61) [5]. Furthermore, another meta-analysis with subtype-specific stratification of glaucoma reported that primary open-angle glaucoma (POAG) was associated with a 29% increased risk of Alzheimer’s disease (relative risk (RR) 1.29, 95% CI 1.16-1.44) [6]. A Japanese cross-sectional study [11] reported that cognitive impairment was more frequent in patients with severe glaucoma (mean deviation (MD) ≤ -12 dB) than in those with mild glaucoma (MD > -12 dB) (OR 2.62, 95% CI 1.01-6.84). Taken together, these findings suggest that reduced visual function itself may contribute to cognitive decline.

Conversely, cognitive impairment may also influence the onset and progression of glaucoma. At present, prospective evidence directly demonstrating that cognitive impairment increases the risk of glaucoma remains limited, and a causal relationship has not been established. However, cognitive dysfunction may lead to poor adherence to topical medications, reduced reliability of visual field testing, and difficulties with postoperative care and follow-up attendance, all of which could indirectly contribute to glaucoma progression. These issues are discussed in greater detail in the following section.

Glaucoma and cognitive impairment also appear to share several pathophysiological features. Previous studies have suggested that abnormal proteins associated with Alzheimer’s disease, such as amyloid-β and tau, which accumulate in the brain, may also be deposited in the retinas of patients with glaucoma [13,14]. Furthermore, elevated levels of Alzheimer’s disease-related proteins, including apolipoprotein E, transthyretin, and α2-macroglobulin, have been reported in the aqueous humor of patients with POAG [12]. In addition, some studies have shown that patients with cognitive impairment exhibit significant thinning of the retinal nerve fiber layer (RNFL) and a larger cup-to-disc ratio (C/D ratio) [15,16]. Moreover, glaucoma and cognitive decline may frequently coexist because both conditions are strongly associated with aging [1-3]. Further studies are needed to clarify the nature of the relationship between these two conditions.

Impact of cognitive impairment on glaucoma management

Reliability of Visual Field Testing

Clinically, cognitive decline can influence glaucoma management in multiple ways. First, cognitive impairment in patients with glaucoma has been associated with a higher frequency of unreliable responses, including increased false-negative (FN) and false-positive (FP) rates in subjective tests such as Humphrey visual field examinations [10,17], which may compromise the accurate assessment of disease progression. A cross-sectional study using the Mini-Cog test demonstrated that patients with cognitive impairment showed higher rates of FN and FP responses in Humphrey visual field testing. Furthermore, poorer performance on the word recall component was strongly associated with increased FN rates [10].

Medication Adherence

Topical medication remains the cornerstone of initial treatment in most patients with glaucoma. However, maintaining adequate adherence to topical therapy remains a major challenge for both patients and healthcare providers. Patients with cognitive impairment, in particular, exhibit poor adherence to anti-glaucoma medications. A population-based case-control study reported that patients with dementia had significantly lower adherence to glaucoma medications compared with those without dementia [18]. Additionally, severe glaucoma has also been associated with reduced medication adherence [7]. Accordingly, in patients with glaucoma and comorbid cognitive impairment, the suitability of topical therapy should be carefully evaluated before initiating treatment.

Surgical Outcomes and Postoperative Management

Patients with cognitive impairment may experience difficulties in attending postoperative follow-up visits and in managing postoperative care after glaucoma surgery. In glaucoma surgery, postoperative management, including eye drop administration, frequent follow-up visits, and additional procedures such as laser suture lysis and needling, requires a certain level of cognitive function. Additionally, a retrospective longitudinal study reported that a higher Charlson Comorbidity Index score, reflecting comorbidity burden including dementia, was significantly associated with the development of postoperative endophthalmitis in patients undergoing glaucoma surgery [19]. Therefore, in glaucoma patients with cognitive impairment, surgical decisions should take postoperative management ability into account. Furthermore, future studies are needed to determine the optimal surgical approach for patients with cognitive impairment.

Cognitive assessment tools in clinical practice

In clinical practice, several cognitive assessment tools are currently used to evaluate cognitive function. In addition, digital application-based approaches, which are expected to have future clinical utility, are also presented in Table 2.

**Table 2:** Cognitive assessment tools applicable in ophthalmology outpatient settings Most conventional cognitive screening tools, such as the MMSE, Mini-Cog, MoCA, and ACE-III, are paper-based instruments. MMSE, Mini-Mental State Examination; Mini-Cog, Mini-Cognitive Test; MoCA, Montreal Cognitive Assessment; ACE-III, Addenbrooke’s Cognitive Examination III; MCI, mild cognitive impairment

Tool	Main cognitive domains	Time	Score/Cut-off
MMSE	Visuospatial ability, language, attention and concentration, working memory, recall, and orientation	5-10 min	30 points; cognitive impairment <24
Mini-Cog	Memory recall, executive function	3-5 min	5 points;
			≤2 suggests cognitive impairment
MoCA	visuospatial/executive function, naming, attention, language, abstraction, memory, and orientation	10-15 min	30 points; <26 suggests MCI
ACE-III	attention and orientation, memory, verbal fluency, language, and visuospatial abilities	~15 min	100 points; <88-82 suggests dementia

Mini-Mental State Examination (MMSE)

The MMSE is one of the most widely used cognitive screening tests worldwide. MMSE has a maximum score of 30 points and is structured around 11 tasks. These tasks evaluate six cognitive domains: visuospatial ability (0-1 points), language (0-8 points), attention and concentration (0-5 points), working memory (0-3 points), recall (0-3 points), and orientation (0-10 points). It requires only 5-10 minutes to administer, making it feasible for routine use. Although the optimal cut-off score of the MMSE remains debated, a threshold of 24 points is generally considered appropriate for cognitive impairment, whereas a higher cut-off of 27-28 points may be more suitable for identifying individuals with mild cognitive decline [20].

Mini-Cognitive Test (Mini-Cog Test)

The Mini-Cog is a brief cognitive screening instrument, comprising three-word recall (0-3 points) and a clock drawing task (0-2 points), and is designed to be completed more rapidly than the MMSE. A score of two or below is generally considered indicative of possible dementia, with reported sensitivity ranging from 76% to 99% and specificity from 83% to 93%, demonstrating a level of validity comparable to that of the MMSE [21].

Montreal Cognitive Assessment (MoCA)

The MoCA is a rapid screening tool developed for the detection of MCI. It was designed to address the limited sensitivity of the MMSE in distinguishing patients with MCI from cognitively healthy older adults. The MoCA requires approximately 10 to 15 minutes to administer, with a total score of 30 points. A score of 26 or above is generally considered within the normal range, whereas scores below 26 suggest the possibility of MCI [22].

Addenbrooke Cognitive Examination (ACE, ACE-R, ACE-III)

The ACE is a questionnaire designed for comprehensive cognitive evaluation, with its most recent version, the ACE-III, now being the most widely applied. The ACE-III encompasses multiple cognitive domains, including orientation, attention and concentration, memory, language, verbal fluency, visuospatial, and perceptual abilities, with a total score of 100 points. It generally requires approximately 15 minutes. For the diagnosis of dementia, recommended cut-off scores are 88 (sensitivity = 1.0; specificity = 0.96) and 82 (sensitivity = 0.93; specificity = 1.0) [23].

Cognitive Screening in Visually Impaired Individuals

Cognitive screening in visually impaired individuals presents important methodological challenges. Many commonly used cognitive screening instruments include visually mediated or graphomotor components, which may lead to FP cognitive impairment. To address this issue, vision-adapted cognitive assessment tools have been developed. The MMSE-blind was designed to assess cognitive function in visually impaired individuals; this modified version (maximum score: 22) excludes items requiring visual processing, including naming, reading, following a written command, writing a sentence, copying, and the three-stage command [24]. Similarly, the MoCA-VI replaces visually dependent items with spoken alternatives and has demonstrated high diagnostic accuracy for dementia and good test-retest reliability [25]. These findings suggest that the use of vision-adapted instruments, or careful interpretation of standard cognitive screening results, is warranted when assessing cognitive function in visually impaired populations.

Digital Application-Based Tools for Cognitive Screening

Recently, various cognitive screening tools for use on tablet devices have been developed, such as MIREVO® (Japan), Cognitive Assessment for Dementia, iPad version 2; CADi2 (Shimane University, Japan), Cogstate Brief Battery (Australia, Cogstate Ltd.), and BrainCheck (USA, BrainCheck Inc.). MIREVO® is a tablet-based application approved as a Software as a Medical Device (SaMD) in Japan. It processes eye-tracking data obtained from task videos using an algorithm and converts them into scores [26]. The test requires approximately three minutes and provides objective results. At present, no studies have applied such digital tools to evaluate cognitive function in patients with glaucoma; however, their use is likely to become valuable in the future.

Current evidence on cognitive function in glaucoma patients

Several studies have used cognitive assessment tools in patients with glaucoma to examine the relationship between glaucoma and cognitive function [27-31]. Most investigations have relied on conventional cognitive screening instruments such as the MMSE, Mini-Cog, and MoCA. One study reported significantly lower MMSE scores in patients with glaucoma than in healthy controls (21.9 ± 7.6 vs 27.7 ± 1.2) [27]. In addition, a large cross-sectional study from Japan found that patients with exfoliation glaucoma (EXG) had lower Mini-Cog scores than those with POAG (4.0 ± 1.0 vs 4.4 ± 0.1) [28]. However, other reports did not identify a significant association between glaucoma and cognitive impairment [27,29]. Although the overall evidence remains inconsistent, cognitive decline may be more evident in patients with advanced glaucoma [11,32]. Therefore, clinicians may consider cognitive assessment, particularly in those with severe disease, to improve treatment adherence and optimize disease management.

Clinical perspectives in the management of glaucoma

The presence of cognitive impairment in patients with glaucoma is not uncommon, particularly among older individuals. A previous meta-analysis demonstrated that the prevalence of cognitive impairment in patients with glaucoma ranged from 12.3% to 90.2%, while the prevalence of dementia ranged from 2.5% to 3.3% [31]. Although its prevalence varies depending on the study population and the screening instruments employed [30,33,34], recent trends suggest a projected increase in the proportion of glaucoma patients with cognitive impairment and dementia, thereby further underscoring the clinical importance of cognitive assessment. MMSE, Mini-Cog, and MoCA are generally simple, require only a short administration time, and are thus considered feasible for use even in busy routine clinical practice; however, their feasibility in routine clinical settings, particularly in ophthalmology, may be influenced by workflow constraints, staff availability, and the impact of visual impairment on test performance. It may be advisable to select cognitive screening instruments with consideration of both the degree of cognitive impairment and the available time for assessment. Furthermore, digital application-based tools are expected to become useful for cognitive assessment in patients with glaucoma in the future.

As previously described, cognitive decline can influence glaucoma management in various ways. Therefore, the assessment of cognitive function in glaucoma care may be clinically relevant, although the current evidence remains largely observational and limited in achieving optimal treatment outcomes. First, patients with glaucoma and cognitive impairment have been reported to show increased rates of FN and FP responses in subjective tests such as Humphrey visual field examinations [10,17]. Thus, careful interpretation is required when evaluating visual field progression. Because conventional subjective visual field testing has inherent limitations, alternative methods that complement these assessments have been developed and are increasingly applied in clinical practice. For example, Koyama et al. [35] developed a three-dimensional convolutional neural network (3D CNN) model to estimate visual field function from optical coherence tomography (OCT) images. This model demonstrated high accuracy and reproducibility through training on large-scale datasets. The significant correlation between estimated and actual visual fields suggests that OCT-based machine learning approaches may serve as a useful adjunctive tool in patients in whom standard perimetry is difficult to perform. Second, cognitive impairment may lead to reduced medication adherence, resulting in suboptimal intraocular pressure control [7-9,18]. Therefore, depending on the severity of cognitive dysfunction, early interventions such as minimally invasive glaucoma surgery (MIGS) or selective laser trabeculoplasty (SLT) may be considered as potential options in selected cases; however, these approaches should be regarded as hypothesis-generating and require further evidence regarding their effectiveness and safety in patients with cognitive impairment. In patients with cognitive impairment, surgical planning should also take into account postoperative management ability and the feasibility of continued follow-up. Given the anticipated increase in patients with cognitive impairment, further studies are required to determine which surgical approaches are safest and most feasible in this population.

## Conclusions

Cognitive impairment, like glaucoma, is an age-related condition. However, cognitive assessment is rarely performed in routine ophthalmic practice. Cognitive screening may be clinically useful in selected older patients with advanced glaucoma, unreliable visual field testing, poor adherence, postoperative care concerns, or suspected cognitive decline. In aging societies, integrating cognitive considerations into glaucoma care may facilitate more individualized and comprehensive management. However, studies directly examining these associations remain limited, and further research is warranted to clarify the relationship between glaucoma and cognitive impairment.
